# Activation of Endogenous FAK via Expression of Its Amino Terminal Domain in *Xenopus* Embryos

**DOI:** 10.1371/journal.pone.0042577

**Published:** 2012-08-06

**Authors:** Nicoletta I. Petridou, Panayiota Stylianou, Neophytos Christodoulou, Daniel Rhoads, Jun-Lin Guan, Paris A. Skourides

**Affiliations:** 1 Department of Biological Sciences, University of Cyprus, Nicosia, Cyprus; 2 Department of Internal Medicine-MMG, University of Michigan Medical School, Ann Arbor, Michigan, United States of America; National Center for Scientific Research Demokritos, Greece

## Abstract

**Background:**

The Focal Adhesion Kinase is a well studied tyrosine kinase involved in a wide number of cellular processes including cell adhesion and migration. It has also been shown to play important roles during embryonic development and targeted disruption of the FAK gene in mice results in embryonic lethality by day 8.5.

**Principal Findings:**

Here we examined the pattern of phosphorylation of FAK during Xenopus development and found that FAK is phosphorylated on all major tyrosine residues examined from early blastula stages well before any morphogenetic movements take place. We go on to show that FRNK fails to act as a dominant negative in the context of the early embryo and that the FERM domain has a major role in determining FAK’s localization at the plasma membrane. Finally, we show that autonomous expression of the FERM domain leads to the activation of endogenous FAK in a tyrosine 397 dependent fashion.

**Conclusions:**

Overall, our data suggest an important role for the FERM domain in the activation of FAK and indicate that integrin signalling plays a limited role in the in vivo activation of FAK at least during the early stages of development.

## Introduction

Cell adhesion and migration are essential processes for embryonic development, wound healing and inflammation. Cell movements and specifically cell migration require coordinated adhesion and detachment of cells from the extracellular matrix (ECM) [1], [2]. The Focal Adhesion Kinase (FAK) is a 125-kDa non-receptor tyrosine kinase that is recruited to focal adhesions and shown to be activated by integrin signalling [3]. As a key mediator of cell-ECM signalling, FAK has an important role in cell adhesion and migration, yet our understanding of the regulation of its activity in these processes remains incomplete [4], [5], [6].

The study of FAK has for a long time primarily focused on its role in cell adhesion and cell migration and as a result a lot of research has been carried out regarding the ways FAK becomes activated downstream of integrin signalling. Upon integrin-mediated adhesion, FAK becomes tyrosine phosphorylated and subsequently activated [7]. Signalling molecules, like Src and phosphatidylinositol 3-kinase (PI3K), are recruited into complexes with FAK, leading to the transduction of biochemical signals that control a wide number of biological processes including cell migration, proliferation, and survival [5], [8], [9]. The involvement of FAK in one or more of these processes is necessary for normal embryonic development, since FAK knockout mice exhibit embryonic lethality [10]. In addition, cells lacking FAK display impaired integrin-dependent cell migration, whereas expression of the dominant negative protein FRNK (FAK Related Non-Kinase) blocks endogenous FAK phosphorylation in vivo and in vitro and suppresses the ability of cells to spread on fibronectin and to elicit integrin-induced signals [10], [11], [12].

FRNK is the C-terminal domain of FAK which contains the focal adhesion targeting (FAT) sequence and the region between the catalytic domain and FAT (a region which contains docking sites for SH3 domain-containing proteins including p130Cas) [11], [13], [14]. The FAT domain has been shown to be both necessary and sufficient for focal adhesion targeting of FAK although the mechanism of focal adhesion targeting has not been fully elucidated [15]. However, focal adhesion targeting has been shown to be necessary for FRNK’s dominant negative activity [16]. FAK contains two additional domains, an N-terminal domain which exhibits homology with FERM domains and a central tyrosine kinase domain [17]. One of the main ways that FAK is regulated is via tyrosine phosphorylation. Several sites of tyrosine phosphorylation have been identified including two tyrosine residues in the activation loop (tyrosines 576 and 577) which regulate its catalytic activity and the major site of autophosphorylation, tyrosine 397 [18], [19]. Tyrosine 397 is located between the catalytic and the FERM domains and in its phosphorylated state serves as a binding site for SH2 domain containing proteins, including Src family kinases as well as PI3K [20], [21]. While the roles of the catalytic and C-terminal domains of FAK have been explored extensively, more recently studies have begun exploring the function of the N-terminal domain in detail.

As mentioned above, the N-terminal domain of FAK exhibits homology with FERM domains, which are structurally conserved domains found in many proteins. The FAK FERM domain has been shown to mediate protein-protein interactions and several binding partners have been identified, including the cytoplasmic tails of the β1 integrin subunit, growth factor receptors and phosphatidylinositol-4,5-bisphosphate (PtdIns(4,5)P_2_–PIP2) [22], [23], [24], [25]. In general, FAK’s FERM domain is primarily viewed as having an inhibitory role on FAK’s activity. Several reports have demonstrated that deletion of the N-terminal domain of FAK leads to elevation of FAK’s catalytic activity, maintaining however responsiveness to integrin signalling [26], [27]. In addition, the FAK FERM domain can bind the FAK kinase domain and can inhibit FAK activity in trans. Specifically, exogenous FERM impairs the catalytic activity of full-length FAK in vitro and FAK signalling in adherent cells in culture [27]. Mutations which partially relieve the FERM binding onto the kinase domain also lead to elevated kinase activity [28]. From the above, a direct auto-inhibitory mechanism for FAK regulation was proposed and the crystal structure of the FERM and kinase domains of FAK support this model. Specifically, the FERM inhibition of FAK’s kinase activity is thought to result from steric inhibition of target protein access to the catalytic cleft and to tyrosine 397 [29]. Release of the FERM binding would presumably allow FAK autophosphorylation on tyrosine 397 and the subsequent recruitment of Src leading to full activation through phosphorylation of the kinase activation loop.

Despite FAK’s importance in development little is known about FAK’s specific role and activation mechanisms in the embryo [30]. In this study, the data presented suggest an important role for the FERM domain in the in vivo activation of FAK during early embryogenesis. Furthermore, we conclude that despite the importance attributed to integrin signalling in the activation of FAK, it appears that integrin signalling plays a limited role in the in vivo setting at least during the early stages of development. This conclusion is supported by two major findings. First, FRNK fails to localize at the plasma membrane where the bulk of phosphorylated FAK is localized and second, expression of FRNK even at very high levels fails to block endogenous FAK phosphorylation in the early embryo. In addition, we show that the FERM domain is both necessary and sufficient for targeting FAK to the plasma membrane. Expression of the FERM domain leads to elevated phosphorylation of endogenous FAK as well as elevated phosphorylation of FAK-Src complex downstream target proteins like p130Cas and paxillin, suggesting that FERM expression can lead to FAK activation. This elevation is dependent on an intact tyrosine 397 site on the N-terminal domain suggesting that FERM activates endogenous FAK through Src.

## Results

### Expression and Phosphorylation of FAK during *Xenopus* Development

**Figure 1:**
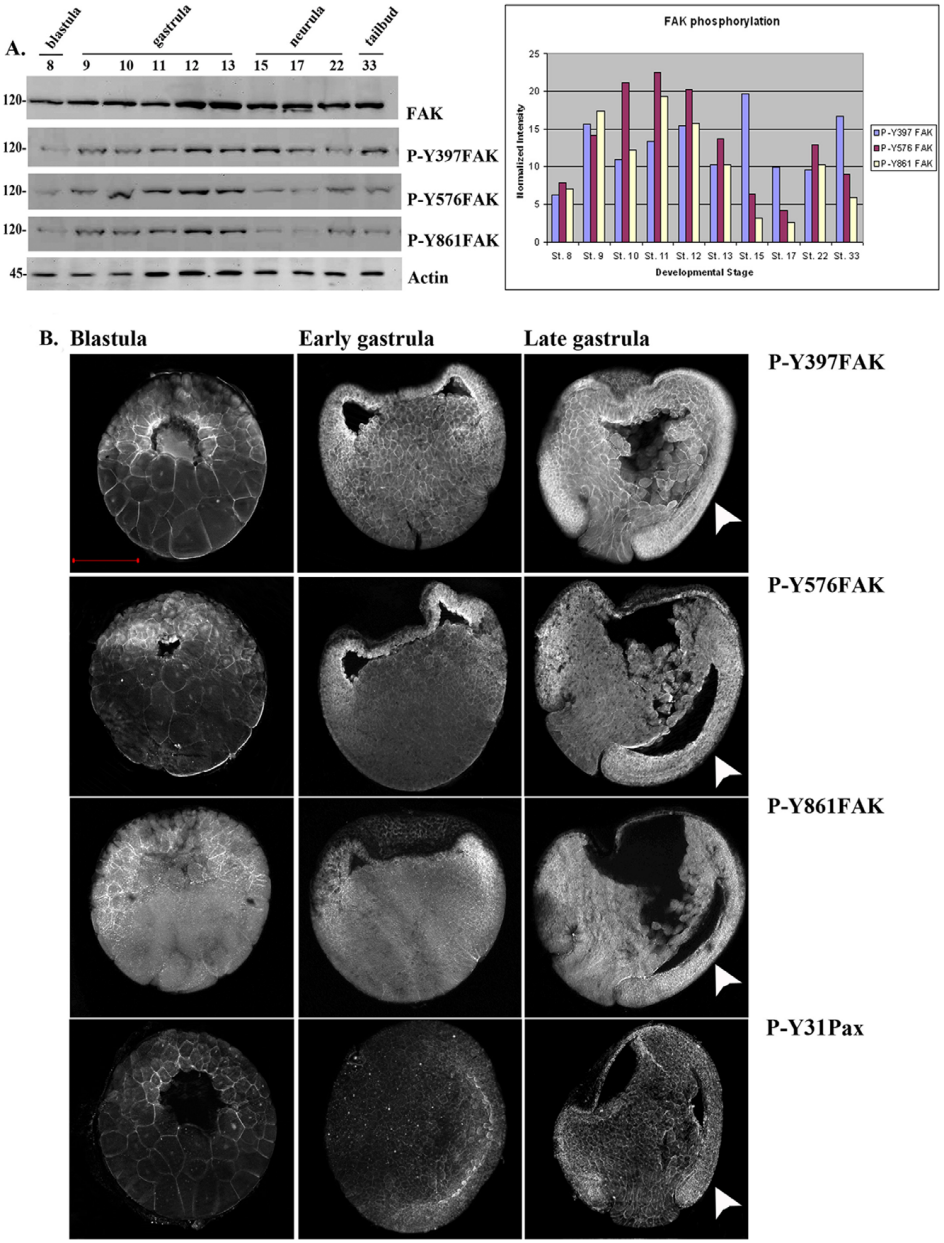
FAK expression and phosphorylation during development. (A) Western Blots from extracts of equal numbers of embryos probed with a monoclonal antibody against the C-terminus of FAK or polyclonal antibodies against the phosphorylated tyrosine residues indicated. FAK is phosphorylated on all three residues both before and after gastrulation. The intensity values from the densitometry analysis of the western blots were normalized against total FAK amount. (B) Blastula (1^st^ column), early gastrula (2^nd^ column) and late gastrula embryos (3^rd^ column) stained with P-Y397, P-Y576, P-Y861 and P-Y31paxillin antibodies as indicated. Phosphorylated FAK and paxillin can be detected on the plasma membrane from early blastula stages including the apical region of superficial blastomeres. During gastrulation elevated levels of phosphorylation are detected in the highly morphogenetic mesodermal tissues (white arrowheads). Scale bar: 400 µm.

**Figure 2:**
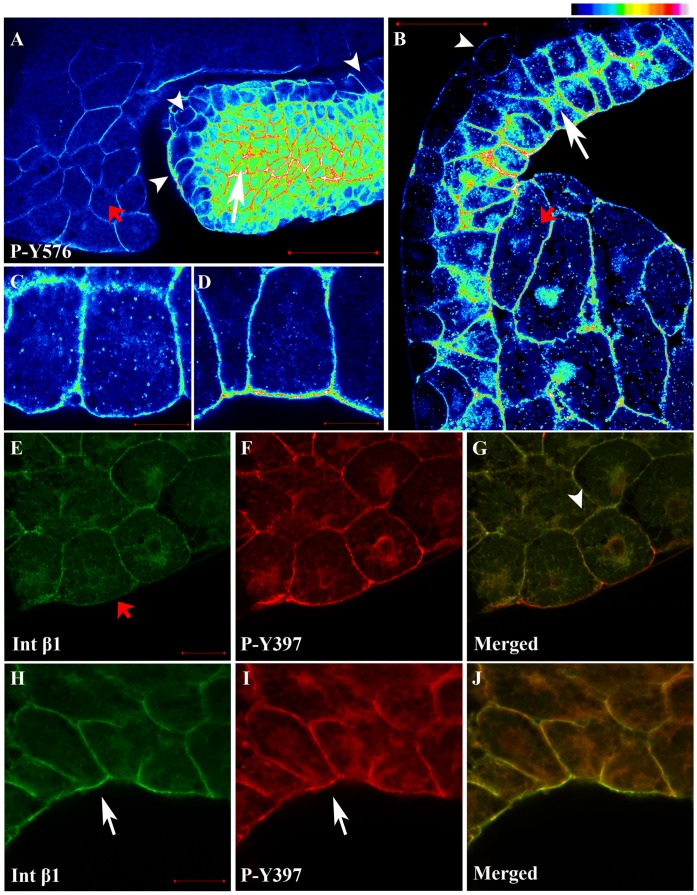
FAK is heavily phosphorylated in mesodermal tissues and integrin-free regions of cells. (A) Intensity color coded confocal section of the dorsal lip region from a whole mount immunostained gastrula stage embryo using a P-Y576 FAK antibody. Mesodermal cells (white arrow) display much higher levels of phospho-FAK than endodermal cells lining the forming archenteron (white arrowheads) and the endodermal cells of the blastopore (red arrow). (B) Same as A but showing the anterior mesendoderm and the animal cap from a whole mount immunostained gastrula stage embryo. The superficial cells of the animal cap (white arrowhead) show lower levels of phospho-FAK signal compared to deep cells (white arrow) and mesendodermal cells (red arrow). (C) High magnification color coded narrow optical section of superficial cells of the animal cap reveals that the apical surface of these cells display similar levels of phospho-FAK compared to the basolateral region while (D) the apical region of the deep cells of the animal cap facing the fibronectin ECM display significantly elevated levels of phospho-FAK compared to the basolateral region. In addition, in the deep cells of the ectoderm labeling of phospho-FAK in the basolateral region is relatively uniform but the apical region displays distinct foci of higher signal intensity (E–G) Confocal optical sections from whole mount immunostained embryos using integrin-β1 (green) and P-Y397 FAK antibodies (red). Integrin-β1 and P-Y397 FAK colocalize on the plasma membrane at cell–cell boundaries (white arrowhead). However phosphorylated FAK is also present on the apical region of the outermost cells of the embryo where integrin-β1 is absent (red arrow). (H–J) Same embryo as above but the cells facing the blastocoel cavity are imaged. Integrin-β1 and P-Y397 FAK colocalize on the apical surface of the cells facing the blastocoel (white arrows). Scale bars: (A) 100 µm, (B) 50 µm, (C, D) 10 µm, (E) 30 µm, (H) 20 µm.

*Xenopus* FAK was originally cloned by Zhang et al. and its expression was analyzed in detail by the DeSimone group. It was determined that FAK is expressed maternally and that elevated levels are found in the highly mophogenetically active mesodermal tissues and in addition at the somitic boundaries. Additionally, increased levels of expression and phosphorylation of FAK were observed during gastrulation indicating that FAK may be involved in regulating embryonic cell adhesive behaviour and morphogenesis [31], [32]. In an effort to better characterize the spatiotemporal expression and phosphorylation of FAK in the embryo we examined the endogenous levels of phosphorylation on tyrosines 397, 576 and 861 in western blotting (Figure 1A) and whole mount immunofluorescence experiments (Figure 1B) using previously characterized antibodies against the phosphorylated forms of the above sites. As shown in Figure 1 all three sites are phosphorylated in all developmental stages we examined including pre-gastrula stages. Phosphorylation of tyrosine 397 follows a similar pattern to what was reported by Hens and DeSimone for total phospho-FAK, ie phosphorylation increases during development with an increase observed during gastrulation [32]. A similar increase is observed for phosphorylation of tyrosines 576 and 861 however for these two sites a drop is observed during neurulation (Figure 1A). Whole mount indirect immunofluorescence shows that phosphorylated FAK is localized at the plasma membrane of the cells (Figure 1B) suggesting that FAK activation takes place at the plasma membrane as expected. Elevated levels of phosphorylated FAK are seen in the highly morphogenetic mesodermal tissues suggesting a possible involvement of FAK in these movements (Figure 1B). Examination of the localization pattern of tyrosine phosphorylated paxillin (Y-31) at these stages reveals a very similar pattern to that of phosphorylated FAK (Figure 1B, 4^th^ row). High magnification optical sections at the blastopore lip reveal that the mesoderm contains much higher levels of phosphorylated FAK (Figure 2A, white arrow) compared to the adjacent endoderm of the blastopore (Figure 2A, red arrow) as well as the single layer of endodermal cells that will line the archenteron and surround the mesoderm (Figure 2A, white arrowheads). In addition, the superficial cells of the ectoderm on the animal cap display lower levels of FAK phosphorylation compared to the deep cells (Figure 2B). The detection of phosphorylated FAK prior to gastrulation including early blastula stages and the presence of phosphorylated FAK on the apical surface of superficial cells is surprising (Figure 1B, 1^st^ column and Figure 2C). Prior to gastrulation there is no fibronectin secretion [33], and no laminin expression [34]. In addition, cells from *Xenopus* embryos are unable to spread or migrate on fibronectin prior to gastrulation [35]. These taken together suggest that there is little, if any cell-ECM signalling at these early stages of development and thus FAK phosphorylation is most likely integrin-independent. This notion is also supported by the presence of phosphorylated FAK at the apical surface of superficial blastomeres which are clearly not exposed to the ECM (Figure 2C). The apical surface of each superficial cell is isolated from the basolateral region with tight junctions which prevent diffusion of membrane bound molecules between the two areas [36]. Thus it is likely that activated FAK at the apical surface of these cells is exclusively activated through mechanisms independent of integrin signalling.

### FAK is Phosphorylated in Integrin-free Regions of the Cell and FRNK Expression Fails to Block FAK Phosphorylation in the Early Embryo

To further explore the possibility that FAK activation in the early embryo is integrin-independent we compared the localization of phosphorylated FAK in relation to that of integrins. Double whole mount in situ immunofluorescence using P-Y397 and integrin-β1 antibodies where carried out and embryos were imaged on a confocal microscope (Figure 2E–J). α5β1-integrin is ubiquitously expressed and is the primary integrin heterodimer responsible for both mesoderm migration on fibronectin as well as control of fibronectin deposition by animal cap cells on the blastocoel roof (BCR) [37]. As seen in Figure 2G phosphorylated FAK is localized tightly on the membrane in a similar fashion to integrin-β1 at sites of cell–cell contact (white arrowhead). However, despite strong phospho-FAK signal on the apical surface of superficial cells, no integrin-β1 staining can be detected in this region of the cell (Figure 2E, red arrow). This is in agreement with previous reports indicating that integrin-α5 is also not detectable on the apical surface of these cells [37]. On the other hand, in cells facing the blastocoel where fibronectin fibrils are assembled [38], [39], both activated FAK and integrins are found on the apical side of the membrane where they colocalize (Figure 2I). Importantly high magnification optical sections from superficial ectodermal cells of the animal cap reveal that the level of FAK phosphorylation is identical between the apical and basolateral regions in these cells suggesting that either FAK activation is completely integrin independent in both cases or that FAK is activated through different mechanisms in the basolateral vs the apical region of these cells (Figure 2C). On the other hand, high magnification optical sections of the deep cells of the ectoderm facing the blastocoel reveals an elevation of FAK phosphorylation at the apical compared to the basolateral region in these cells (Figure 2D). This could be interpreted as integrin based elevation since fibronectin is lining the blastocoel and both α5 and β1 integrins are present in this region [37], [40]. It should also be noted that in these high magnification images of deep ectodermal cells the phospho-FAK signal in the basolateral region appears relatively uniform and no focal adhesion like structures can be detected. However on the apical surface of these cells which is facing the fibronectin network of the BCR, the phospho-FAK staining is of higher intensity with clearly visible high intensity foci which may represent focal adhesion like structures (Figure 2D).

**Figure 3:**
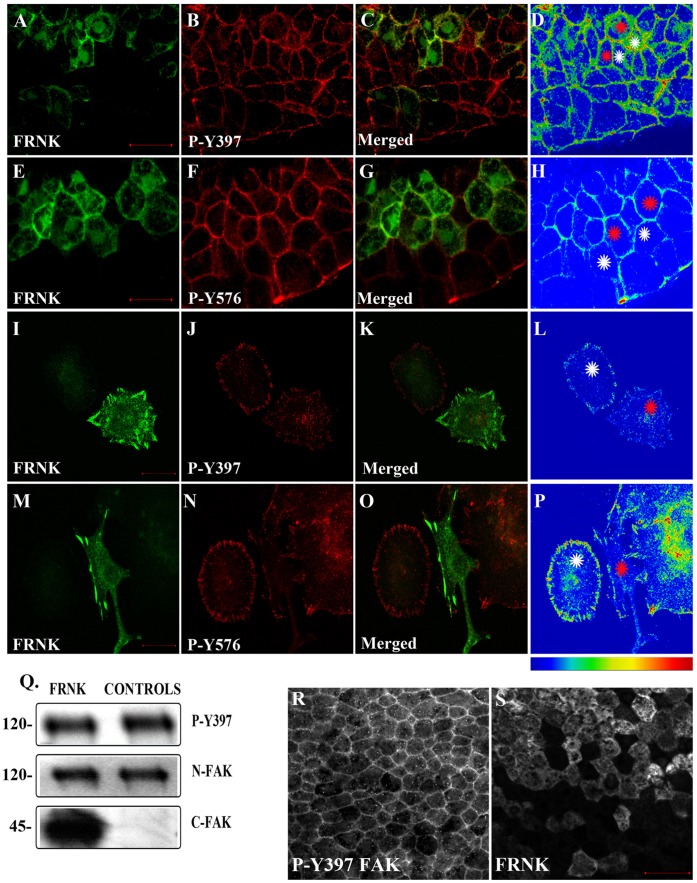
FRNK does not act as a dominant negative in early *Xenopus* embryos. (A–D) Optical sections of whole mount immunostained embryos injected with 1 ng GFP-FRNK at the two dorsal blastomeres at the four-cell stage. Embryos were stained with anti-GFP (A) and anti-P-Y397 (B). C is the merged image and D an intensity color coded image of the anti-P-Y397 signal. FRNK injected cells are indicated with red stars and control cells with white stars. FRNK expression fails to reduce the phosphorylation levels of endogenous FAK on tyrosine 397. (E–H) Same as A–D, but the embryos were stained with anti-GFP (E) and anti-P-Y576 (F). FRNK expressing cells display similar levels of phosphorylation on tyrosine 576 as neighboring control cells. (I–L) Confocal images of A6 *Xenopus* cells transfected with GFP-FRNK. Cells were stained with anti-GFP (I) and anti-P-Y397 (J). K is the merged image and L an intensity color coded image of the anti-P-Y397 signal. FRNK expression leads to reduction of the phosphorylation levels of FAK on tyrosine 397 at the focal adhesions. (M–P) Same as I–L but the cells were stained with anti-GFP (M) and anti-P-Y576 (N) antibodies. FRNK expression leads to downregulation of the endogenous phosphorylation levels of FAK on tyrosine 576 at the focal adhesions. (Q) Western blot analysis of control and injected gastrula stage embryos with 1 ng FRNK at the animal pole of both blastomeres of two cell stage embryos. FRNK expression fails to reduce endogenous FAK phosphorylation on tyrosine 397. FRNK expression was verified using a FAK antibody raised against the C-terminus of the protein. (R–S) Localization of P-Y397 FAK (R) and FRNK (S) in animal pole cells of stage 10 *Xenopus* embryos. P-Y397 FAK shows strong membrane localization while FRNK is primarily cytoplasmic in these cells. Scale bars: (A) 40 µm, (E) 30 µm, (I) 20 µm, (M) 20 µm, (R–S) 40 µm.

To further examine the integrin component of FAK activation in the early embryo we overexpressed FRNK and examined its effect on the endogenous phosphorylation of FAK on tyrosines 397 and 576. Overexpression of FRNK has been shown to block cell spreading and tyrosine phosphorylation of endogenous FAK, paxillin, and tensin [41]. Although focal adhesion localization and thus integrin colocalization of FRNK is not the sole determinant for its dominant negative activity, it is required for this dominant negative activity suggesting that FRNK exerts its effect by blocking FAK specifically at integrin based activation sites [16]. However, as shown in Figure 3 expression of FRNK in DMZ cells has no effect on the levels of phosphorylation of FAK on tyrosines 397 and 576 during gastrulation in vivo as determined by immunofluorescence and western blotting (Figure 3 A–H, Q). This is in contrast to the clear downregulation of phosphorylation on both tyrosine 397 and 576 seen when we expressed FRNK via transfection in the *Xenopus* A6 cell line (Figure 3I–L, M–P respectively). In agreement with the lack of dominant negative activity FRNK fails to localize at the plasma membrane in animal cap cells unlike phosphorylated endogenous FAK which is found exclusively on the plasma membrane in these cells (Figure 3R, S). These results demonstrate a strong context dependence with regards to the dominant negative activity of FRNK and are in agreement with the possibility that the bulk of phosphorylated FAK in the early embryo results through integrin independent mechanisms.

### The FERM Domain is both Necessary and Sufficient for Plasma Membrane Localization of FAK in Integrin-free Regions of the Cell

**Figure 4:**
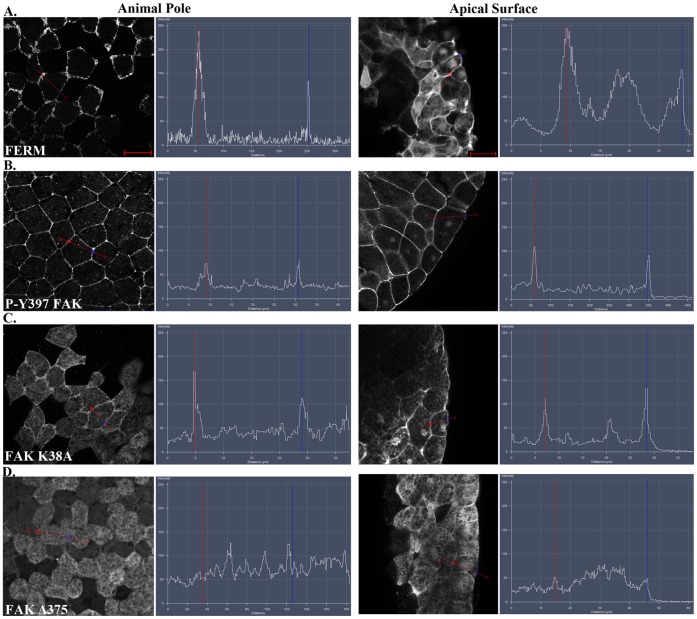
The FERM domain is necessary and sufficient for membrane localization of FAK at integrin-free regions. Confocal images and intensity profiles of the indicated constructs after whole mount immunostaining. The first column are top views of superficial cells of the animal cap in intact embryos and the second column are views from sagittally sectioned embryos that reveal the localization of each construct on the apical surface of superficial cells. Apical region of superficial blastomeres is to the right. (A) The FERM domain shows strong plasma membrane localization in the top view and is strongly localized to the apical surface. (B) Endogenous phosphorylated FAK shows very strong plasma membrane localization in the top view and is localized on the basolateral and apical surface of the cell. (C) Full length FAK with the point mutation K38A exhibits strong membrane localization. (D) Deletion of the FERM domain (HA-Δ375 FAK construct) abolishes the plasma membrane localization of FAK. Scale bars: 25 µm.

The above data raised the possibility that FAK is primarily activated through integrin-independent mechanisms in the early *Xenopus* embryo. Since the C-terminus of FAK which is both necessary and sufficient for focal adhesion localization fails to localize at the plasma membrane of animal cap cells (Figure 3S) we postulated that the N-terminus which has been shown to bind PIP2 and growth factor receptors may in fact be the major determinant for the localization of active FAK in vivo [4], [5], [6]. To explore the role of the FERM domain in the localization of endogenous FAK on the plasma membrane, a series of constructs were generated (based on chicken FAK which shares a 91% identity and 95% similarity at the amino acid level with *Xenopus* FAK and conservation of all tyrosine phosphorylation sites) and examined with respect to their localization in cells of the animal pole and their ability to specifically localize to the integrin-free apical surface of these cells. Each construct was expressed as an HA tagged protein through mRNA injection at the two AP-dorsal blastomeres of four-cell stage embryos, and embryos were subsequently processed for whole mount immunofluorescence using a monoclonal anti-HA antibody. As shown in Figure 4 the FERM domain, unlike FRNK, displays strong plasma membrane localization and is also found on plasma membrane associated vesicles (Figure 4A). This pattern closely matches that of phosphorylated FAK (Figure 4B) suggesting that the FERM domain rather than the FAT domain is responsible for membrane localization of active FAK in the embryo. To further investigate the role of the FERM domain in the localization of activated FAK we examined the localization of the full length FAK K38A point mutant, in which the FERM kinase domain interaction is compromised, and compared it to that of the Δ375 mutant, which lacks the FERM domain. Both constructs are constitutively active due to the loss in the case of the K38A mutant of the FERM kinase inhibitory interaction and absence of the FERM domain in the case of the Δ375. The two constructs exhibit significant differences in terms of their ability to localize to the plasma membrane with the K38A exhibiting strong membrane localization while the Δ375 is very diffuse and appears completely absent from the plasma membrane in the cells of the animal cap (Figure 4C and D respectively).

These results suggest that the FERM domain is both necessary and sufficient for the localization of FAK on the plasma membrane.

### Expression of the N-terminal Domain of FAK Leads to Elevated Phosphorylation of Endogenous FAK and Downstream Targets in a Src Dependent Manner

**Figure 5:**
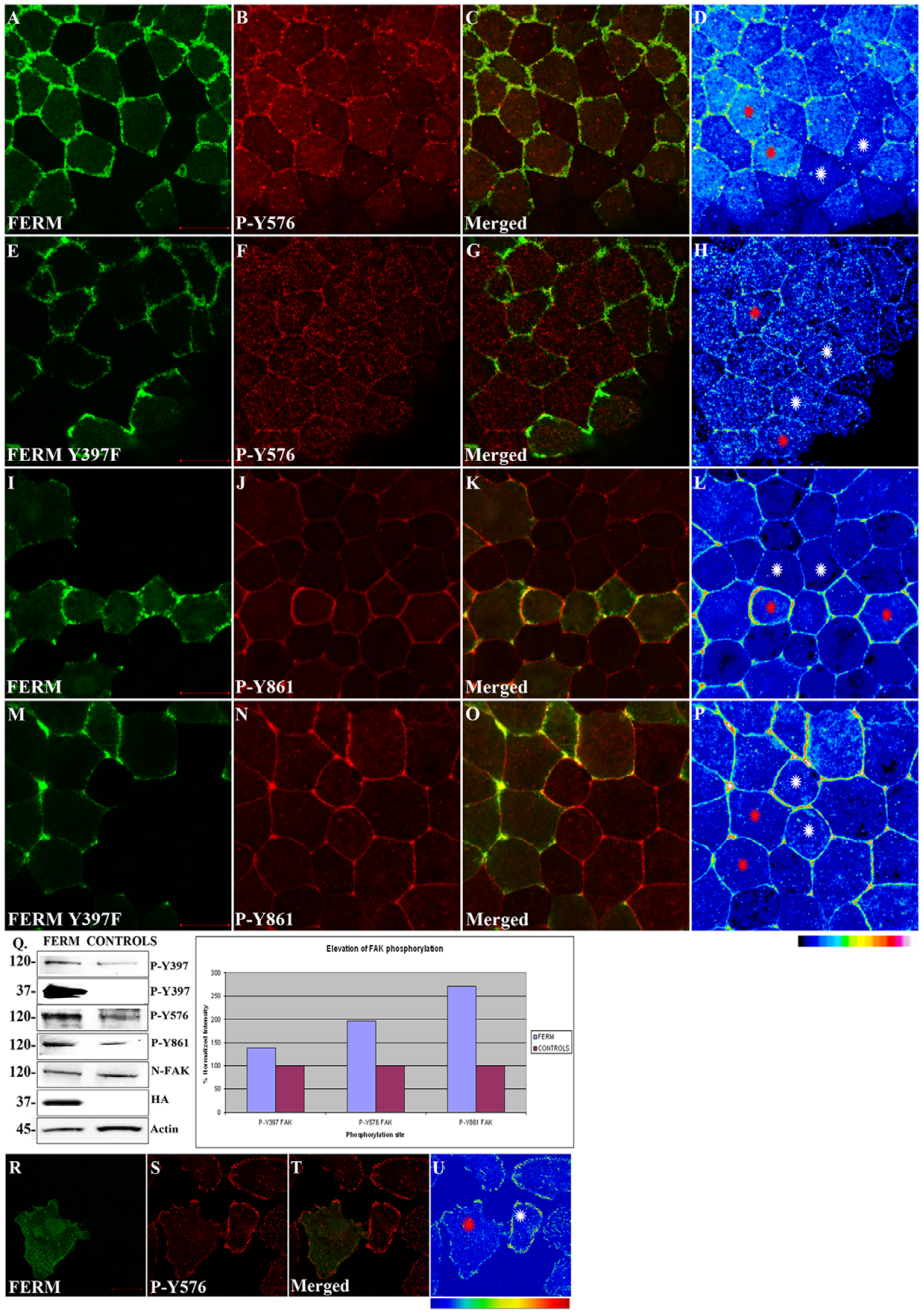
The FERM domain leads to activation of endogenous FAK in a tyrosine 397 dependent manner. HA-FERM and HA-FERM Y397F injected embryos in one blastomere at the animal pole of two cell stage embryos were processed for whole mount immunostaining using an HA antibody (green) to reveal expressing cells and the respective phospho-specific antibodies (red) as indicated. In each case individual signals for each secondary are shown in addition to a merged image and finally an intensity color coded image of the respective phospho-specific antibody signal. HA-FERM and HA-FERM Y397F injected cells are indicated with red stars and un-injected cells with white stars. (A–D) Levels of phosphorylated tyrosine 576 are elevated in HA-FERM overexpressing cells compared to controls. (E–H) Overexpression of HA-FERM Y397F has no effect on the endogenous levels of phosphorylated tyrosine 576. HA-FERM Y397F expressing cells have the same levels of phosphorylated endogenous FAK on tyrosine 576 with neighboring control cells. (I–L) Levels of phosphorylated tyrosine 861 are elevated in HA-FERM expressing cells compared to controls. (M–P) Overexpression of HA-FERM Y397F has no effect on the endogenous levels of phosphorylated tyrosine 861. HA-FERM Y397F expressing cells have the same levels of phosphorylated endogenous FAK on tyrosine 861 with neighboring control cells. (Q) Total lysates from HA-FERM injected gastrula stage embryos contain comparable levels of endogenous FAK as un-injected controls but elevated levels of phosphorylated FAK on tyrosines 397, 576 and 861. Blotting using the anti-P-Y397 antibody shows that the exogenously expressed FERM is heavily in trans phosphorylated on tyrosine 397 (2nd row). The intensity values from the densitometry analysis were normalized against total FAK and present the average increase in phosphorylation from three independent experiments (R–U) Confocal images of A6 *Xenopus* cells transfected with HA-FERM. Cells were stained with anti-HA (R) and anti-P-Y576 (S). T is the merged image and U an intensity color coded image of the anti-P-Y576 signal. Transfected cells are shown with red stars and controls with white stars. HA-FERM transfected cells show reduced levels of tyrosine 576 phosphorylation suggesting that FERM expression blocks FAK activation in these cells. Scale bars: (A) 40 µm, (E) 30 µm, (I) 20 µm, (M) 50 µm, (R) 20 µm.

To further explore the role of the FERM domain in the activation of FAK in *Xenopus* we examined the effects of FERM domain overexpression in the developing embryo. Embryos were injected with the FERM domain either at the animal pole or at the two dorsal blastomeres of four cell stage embryos and were allowed to develop to tadpole stages. FERM expressing embryos developed normally and were identical to controls suggesting that FAK function was not being affected (data not shown). To examine the effects of FERM expression on endogenous FAK the experiment was repeated and embryos were either fixed or lysed at gastrula stage. As shown in Figure 5, cells expressing FERM, show elevated levels of endogenous phosphorylated FAK on tyrosines 576 and 861 compared to un-injected neighbouring cells suggesting that FERM expression leads to activation of endogenous FAK (Figure 5A–D, 5I–L respectively, red stars: injected cells, white stars: control cells). This is a surprising finding and several experiments were carried out to ensure that the phospho-FAK antibodies used were in fact specific in this context. These control experiments are described in detail in the methods section and presented in Figure S1. To confirm the activation of FAK in FERM overexpressing embryos western blotting experiments and densitometry analysis were carried out. Lysates from FERM injected embryos contain comparable levels of total FAK as controls but elevated phosphorylation on tyrosines 397, 576 and 861 (Figure 5Q). It should be noted that only a subset of the cells in the embryo are expressing the construct (∼50%) so the upregulation is effectively underestimated in western blotting experiments. In addition blotting of the HA-FERM with an anti P-Y397 antibody shows that the exogenously expressed protein is phosphorylated on tyrosine 397 in agreement with previously published work (Figure 5Q, 2^nd^ row) [25], [42]. Expression of the N-terminus of FAK has been previously shown to block integrin-dependent FAK activation [27], [28]. To preclude the possibility that the observed effect is *Xenopus* specific we expressed the FERM domain in *Xenopus* A6 cells and examined the effects on endogenous levels of phospho-576 via indirect immunofluorescence. As shown in Figure 5 (R–U) FERM expression in *Xenopus* adherent cells results in a moderate reduction (compared to the more drastic effects of FRNK expression, Figure 3M–P) of phospho-576 levels indicating that FERM does in fact block FAK activation in cultured *Xenopus* cells. The opposite results obtained in vivo and in vitro with regard to the effects of FERM expression may be explained by a differential effect of FERM expression in integrin vs non integrin-based activation of FAK. Expression of the N-terminus of FAK in FAK null cells has been shown to actually partially rescue the EGF induced cell migration defect suggesting that the FERM domain can partially transduce growth factor based signals autonomously supporting this possibility [25].

**Figure 6:**
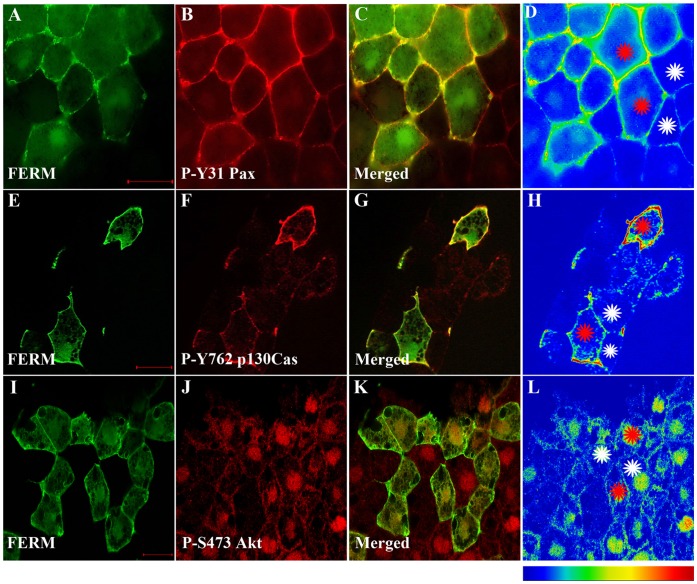
The FERM domain activates endogenous FAK leading to increased phosphorylation of FAK/Src targets. (A–D) Embryos injected with HA-FERM mRNA in two blastomeres, at the animal pole, at the four cell stage were processed for immunofluorescence using anti-HA (green) and anti-P-Y31 paxillin (red) antibodies. C is the merged image and D is an intensity color coded image. FERM expressing cells display elevated levels of phosphorylated paxillin (red stars) when compared with un-injected neighbouring cells (white stars). (E–H) Same as (A–D) but comparing phosphorylation levels of p130Cas on tyrosine 762 between FERM expressing and control cells. FERM expressing cells show elevated levels of phosphorylated p130Cas (red stars), when compared with un-injected cells (white stars). (I–L) Same as (A–D) but comparing levels of phosphorylated Akt on serine 473 between FERM expressing and control cells. Levels of phosphorylated Akt are comparable in FERM expressing cells to those of control neighboring cells. Scale bars: 20 µm.

Since tyrosine 397 is a known Src binding site [21] and tyrosines 576 and 861 are targets of Src [18], [43] we went on to examine the possibility that an intact tyrosine 397 was required for the FERM induced activation of FAK. We generated a FERM Y397F construct and the construct was overexpressed in *Xenopus* embryos. As shown in Figure 5, cells expressing FERM Y397F do not display elevated levels of phosphorylation on tyrosines 576 and 861 (Figure 5E–H and M–P) suggesting that the observed activation of endogenous FAK is dependent on Src. This is also supported by the fact that in embryos treated with the Src inhibitor PP2, tyrosine phosphorylation on both 576 and 861 is severely reduced indicating that, in the developing embryo, FAK phosphorylation of the above residues is dependent on Src (Figure S1). Finally, to confirm the FERM induced activation of endogenous FAK we examined the phosphorylation status of p130Cas and paxillin, two FAK-Src downstream targets [44], [45]. Expression of FERM, but not FERM Y397F, leads to elevated phosphorylation of both paxillin and p130Cas but not of Akt which is a substrate of PI3K (Figure 6A–D, E–H, I–L) [46]. These results show that FERM expression leads to activation of endogenous FAK and subsequent phosphorylation of FAK-Src targets in a tyrosine 397 dependent manner.

## Discussion

The Focal Adhesion Kinase is a cytoplasmic kinase shown to be involved in a number of diverse processes including cell adhesion, migration, proliferation and survival. It has also been shown to be necessary for embryonic development since FAK knockout mice die early during development due to defects of the axial mesoderm [10]. FAK as a focal adhesion protein has been primarily studied with respect to integrin-based activation on 2D matrices. On such matrices it has been shown to be activated downstream of integrin clustering and to have an important role in the assembly and disassembly of focal adhesions [47], [48], [49], [50], [51], [52]. However, focal adhesions are much smaller on soft matrices and different in structure, localization, and function in cells embedded in 3D matrices which resemble the in vivo setting better [53], [54], [55]. In addition cell polarization is also dependent on matrix rigidity and controlled by mechanosensing at the focal adhesions [55]. FAK’s phosphorylation is much lower in cells grown on soft matrices and surprisingly no tyrosine 397 phosphorylation can be detected on 3D-matrix adhesions [56], [57], [58]. In fact downregulation of some proteins has the opposite effect in terms of their role in cell migration when tested on 2D vs 3D matrices. Overall it appears that regulation of 2D cell motility by focal adhesion proteins is not necessarily predictive of regulation of cell motility in a 3D matrix [53]. Things get even more complicated when examining focal adhesion proteins in the context of a living organism in which case not only the cell is faced with a 3D matrix but it also faces a 3D cell–cell adhesion network. These differences raise the need for the study of adhesion molecules like FAK in an in vivo setting in order to allow the integration of the valuable knowledge generated on FAK signaling in vitro back to a more physiologically relevant context.

Here we explore the activation of the Focal Adhesion Kinase in the context of the *Xenopus* embryo. We initially examined FAK phosphorylation on three major tyrosine residues 397, 576 and 861 during development. In agreement with previously published work, we observed elevated FAK phosphorylation on all three residues during gastrulation suggesting a possible role of FAK in embryonic morphogenesis. Specifically, phospho-FAK levels were elevated in the mesoderm compared to the endoderm in gastrula stage embryos and deep cells of the animal cap displayed elevated levels of phospho-FAK compared to the cells of the outermost epithelium. Surprisingly, phosphorylation was detected on all three residues from early blastula stages before the mid-blastula transition and well before the initiation of gastrulation and cell movements. What the role of FAK during these early developmental stages might be is not known but the mechanism of activation is not likely to be through integrins but rather through growth factor receptors. While FAK could be detected in the cytosol the plasma membrane and the nuclei of cells in the embryo, tyrosine phosphorylated FAK was only found on the plasma membrane suggesting that activation takes place there. No focal adhesion like structures could be detected and the staining appears to be uniform on the surface of these cells. However at gastrula stages and specifically in the deep cells of the ectoderm which are in contact with the fibronectin matrix of the BCR, phospho-FAK is elevated and displays foci of higher signal intensity that resemble focal adhesion like structures. On the other hand, in superficial cells of the ectoderm we detected equal levels of phosphorylated FAK on the apical surface of the plasma membrane compared to the basolateral regions. Since the apical region of the plasma membrane is free from integrins and isolated from the basolateral with tight junctions this supports the notion that FAK activation during early development can be integrin-independent. Another possibility is that FAK activated at the basolateral region of these cells diffuses through the cytosol and relocalizes on the apical side of the plasma membrane. This is unlikely though because this would presumably generate a gradient of higher levels of phospho-FAK at the periphery of the apical membrane vs the center. This does not appear to be the case since the intensity of phospho-FAK signal is uniform on the apical plasma membrane of superficial cells.

We go on to show that FRNK, which has been shown to act as a dominant negative and block integrin-based FAK activation, fails to reduce FAK activation in the embryo, as determined by undiminished levels of FAK’s tyrosine phosphorylation on key residues including tyrosine 397 and 576 [44]. We have in the past shown that FRNK does in fact reduce endogenous FAK phosphorylation of mesodermal explants plated on fibronectin and show now that it can do the same in *Xenopus* cell lines [59]. In all these cases however, FAK phosphorylation primarily derives from cell-ECM adhesion, whereas we present evidence suggesting that this is not the case in the early embryo. In fact the inability of FRNK to act as a dominant negative could itself be considered indirect evidence that FAK is activated independently of integrins in this context. In addition FRNK, despite containing the FAT domain which has been shown to be both necessary and sufficient for targeting FAK to focal adhesions, fails to target FAK at the plasma membrane in the embryo and is specifically absent from the integrin-free apical region of the plasma membrane in superficial blastomeres. This inability may explain why it fails to block FAK activation since it is presumably unable to compete endogenous FAK off of its complexes at the plasma membrane. FRNK has also been shown to act as a dominant negative during somitogenesis in *Xenopus* tadpoles. Specifically, FAK and other focal adhesion molecules, like paxillin as well as fibronectin and integrins, have been shown to localize at the intersomitic boundaries leading to the conclusion that focal adhesion contacts mediate the stabilization of somite boundaries [60], [61], [62]. Since FAK is activated through integrins in this context these data confirm that FRNK is able to block integrin-based activation of FAK in vivo. Although FRNK has been shown to block GFR based activation of FAK in cultured cells, for example PDGF induced activation of FAK in Vascular Smooth Muscle Cells is blocked by FRNK expression, it is possible that in this context where PDGF also induces the migration of these cells, FAK’s activation is still largely integrin-dependent and indirect [63]. In addition, the FAT domain in FRNK has been shown to be the major determinant for FRNK’s dominant negative function [41] suggesting that targeting to integrin-based complexes is the mechanism through which FRNK acts, suggesting that in the absence of the ability to target non integrin-based FAK complexes FRNK would not be able to act in a dominant negative fashion.

Exploring the domains of FAK that might be responsible for targeting of FAK to integrin-free regions of the plasma membrane we found that the N-terminal region of FAK is both necessary and sufficient for membrane localization. Exogenously expressed FERM recapitulates the localization of tyrosine phosphorylated FAK while FRNK fails to do so. In addition, deletion of the FERM domain leads to the reduction of plasma membrane localization of full length FAK. Overall these data suggest that the major determinant for localization of FAK on the plasma membrane of superficial cells of the embryo is the N-terminus. However, it should be noted that in DMZ cells which display the highest levels of FAK phosphorylation during gastrulation neither the FERM domain nor the FAT domain are sufficient to strongly target FAK to the plasma membrane (Figure S2) suggesting that in these cells both the FAT and the FERM domain may cooperate to target active FAK on the plasma membrane.

The fact that exogenously expressed FERM has such a dramatically different localization compared to full length FAK is most likely due to the fact that the majority of FAK in the cell is in the closed conformation with the FERM domain unavailable to bind growth factor receptors and PIP2 [64]. Expressed autonomously it no longer is impeded by its interaction with the kinase domain and free to bind targets on the plasma membrane. The strong membrane localization of the FERM domain coupled with the fact that it was shown to block FAK activation in trans raised the possibility that exogenously expressed FERM could block endogenous FAK activation in vivo. However, FERM expression lead to activation of endogenous FAK in a tyrosine 397 dependent fashion. Exogenous FERM is heavily phosphorylated on tyrosine 397 at the plasma membrane, presumably in trans by active endogenous FAK which is also at the plasma membrane. The fact that tyrosine 397 is necessary for the FERM induced activation of endogenous FAK leads to the conclusion that the exogenous phosphorylated FERM recruits Src to the plasma membrane leading to additional FAK phosphorylation. It is possible that the FERM domain in the context of non integrin-based activation has a greater affinity for the plasma membrane (PIP2 and GFRs) than for the kinase domain of endogenous FAK and thus fails to block endogenous FAK in trans but rather leads to further activation via Src. This interpretation is in agreement with recent data showing that Src is required for PDGF dependent activation of FAK whereas FAK is actually necessary for Src activation at integrin-based adhesions [65]. In addition, FERM domain expression has been shown to partially rescue the EGF stimulated migration defect of FAK null cells. This coupled with the fact that the FERM domain can autonomously interact with EGFR suggests that the FERM domain can in fact promote GFR based FAK signaling while blocking integrin-based activation [27], [28]. The fact that FERM expression leads to FAK activation rather than downregulation in the early embryo supports the notion that FAK activation in this context is largely integrin-independent.

The results presented in this paper suggest that FAK is activated primarily through integrin-independent mechanisms in the early embryo and that the FERM domain and not the FAT domain is the primary determinant for FAK’s localization at the plasma membrane, at least in integrin-free regions of the cell. In addition, the data suggest an important role of the FERM domain in the in vivo activation of FAK and provide new insights regarding the differences between integrin and GFR activation of FAK. Finally, these experiments suggest kinase dependent roles for FAK, which are independent of cell movement and cell-ECM interactions, very early during development. The generation of a dominant negative mutant that enables inhibition of FAK activity in early embryos would be an important step in order to begin exploring the possible roles of FAK at these early embryonic stages.

## Methods

### Embryos, Explants and Microinjections

*Xenopus laevis* embryos from induced spawning were staged according to Nieuwkoop and Faber (1967). Operation techniques and buffers have been described [66]. *Xenopus* embryos were fertilized in vitro and dejellied using 1.8% L-cysteine, pH 7.8, then maintained in 0.1X Marc’s Modified Ringer’s (0.1X MMR). Microinjections were performed in 4% Ficoll in 0.33X MMR. The embryos were injected with mRNA at the 2 and 4-cell stage according to established protocols [67]. The injections amounts per embryo were the following: GFP-FRNK 500 pg –1 ng, HA-FAK 100–200 pg, HA-FERM 500 pg, HA-FERM Y397F 500 pg, HA-FAKΔ375 500 pg, HA FAK K38A 300 pg. After the injections the embryos were cultured in 4% Ficoll in 0.33X MMR until stage 8 and then cultured in 0.1X MMR at room temperature.

### Cell Culture and Transfections

The *Xenopus* cell line A6 [68] was grown in L-15 medium Leibovitz plus 10% FCS at room temperature. Transfection of A6 cells with the constructs HA-FRNK pCS108 and HA-FERM pCS108 was performed using Lipofectamine (Lipofectamine 2000, Invitrogen, UK) according to the manufacturer’s protocol. The NIH3T3 (ATCC) and FAK^−/−^ (ATCC) cell lines were grown in DMEM medium plus 10% FBS at 37°C. These cell lines were used in immunofluorescence experiments for the specificity of the phospho antibodies.

### Antibodies and Surface Labelling

Indirect immunofluorescence assays were carried out as described [69], [70] with modifications. Transfected and control A6 *Xenopus* cells were plated on glass coverslips (charged with HCl) washed three times with phosphate buffered saline (PBS) containing 0.5 mM MgCl_2_ and 0.5 mM CaCl_2_ (PBS++) and then fixed for 10 min in 4% paraformaldehyde solution in PBS. Fixation was followed by addition of 50 mM glycine solution in PBS and then the cells were permeabilized using 0.2% Triton-X solution in PBS for 10 min. Permeabilized cells were blocked using 10% normal donkey serum (Jackson Immunoresearch, USA) for 30 min. Cells were incubated with primary antibodies diluted in 10% normal donkey serum solution in PBS for one hour. The primary antibodies used were P-Y397 FAK rabbit polyclonal (1∶500, 44624G-Invitrogen, UK) or P-Y576 FAK rabbit polyclonal (1∶500, 44652G-eBiosource Invitrogen, UK) in combination with HA mouse monoclonal (1∶500, sc-7392-Santa Cruz, USA). Cells were then washed five times in PBS. Secondary antibodies used were Cy3 anti-rabbit (1∶500, 711-165-152, Jackson Immunoresearch, USA) and Alexa 488 anti-mouse (1∶500, A11029, Molecular probes Invitrogen, UK).

For whole mount immunostaining, embryos were fixed in 10% 10XMEMFA-0.1 mM MOPS pH 7.4, 2 mM EGTA, 1 mM MgSO_4_, 3.7% formaldehyde-10% formaldehyde and 80% water for 2 hours at room temperature and the vitelline envelope was removed manually. Embryos were then bisected and permeabilized for at least 5 hours in 1XPBS, 0.5% Triton, 1% DMSO (Perm solution) and blocked for 2 hours in 10% Normal Goat or Normal Donkey serum in Perm solution. Embryos were then incubated with primary antibodies. These included P-Y397FAK rabbit polyclonal (1∶2000, ab4803-Abcam, USA), P-Y397 FAK mouse monoclonal (1∶500, MAB1144-Chemicon Millipore, USA), P-Y397 FAK rabbit polyclonal (1∶500, 44624G-Invitrogen, UK), P-Y576 FAK rabbit polyclonal (1∶500, 44652G-eBiosource Invitrogen, UK), P-Y576 FAK rabbit recombinant monoclonal (1∶500, 700013-Invitrogen, UK), P-Y576 rabbit polyclonal (1∶1500, sc-16563-R, Santa Cruz), P-Y861 FAK rabbit polyclonal (1∶500, 44-626G-eBiosource Invitrogen, UK), P-Y31 Paxillin rabbit polyclonal (1∶150, sc-14035-Santa Cruz, USA), P-S473 Akt rabbit polyclonal (1∶300, sc-7985-Santa Cruz, USA), HA mouse monoclonal (1∶100, sc-7392-Santa Cruz, USA), HA rabbit polyclonal (1∶500, NB600-363-Novus, UK). The incubation was performed overnight at 4°C. Embryos were then washed four times in Perm solution for 20 min, incubated for 2 hours RT with secondary antibodies Alexa 488 anti-mouse (1∶500, A11029, Molecular probes Invitrogen, UK), Alexa 488 anti-rabbit (A11034, Molecular probes Invitrogen, UK), Cy3 anti-mouse (1∶500, 715-165-150, Jackson Immunoresearch, USA), Cy3 anti-rabbit (1∶500, 711-165-152, Jackson Immunoresearch, USA) at RT and then washed four times in Perm solution for 20 min. Clearing of embryos was performed by immersing the embryos in two parts Benzyl Benzoate and one part Benzyl Alcohol after dehydration (Murray’s Clearing Medium).

The phosphospecific FAK antibodies although previously characterized used in the figures were tested further for specificity in the context they were used. Two-cell stage embryos were treated with 20 µΜ of the Src inhibitor PP2 (P0042, Sigma) until gastrula stages in order to block phosphorylation of endogenous FAK. Use of the Src inhibitor led to dramatic reduction of the signal of both the P-Y576 and P-Y861 in gastrula stage embryos (Figure S1A). In addition, both the P-Y397 and P-Y576 antibodies which were used extensively were tested in FAK knockout cells where they fail to detect focal adhesions in immunofluorescence experiments (Figure S1B and data not shown). To ensure that neither the P-Y576 nor the P-Y861 antibodies bind exogenous phosphorylated FERM western blotting experiments were carried out where FERM was initially probed with either a P-Y576 or a P-Y861 antibody, then striped and reprobed using a P-Y397 antibody. Neither P-Y576 nor P-Y861 bind exogenous FERM (Figure S1C). Moreover, colocalization analysis was performed in HA-FERM expressing embryos double stained for HA and P-Y576 or P-Y397. Although HA colocalizes strongly with P-Y397 as expected it does not with anti P-Y576 suggesting that no cross reactivity or bleed through is present (Figure S1D). Additionally, 30 ng of FAK morpholino (TTGGGTCCAGGTAAGCCGCAGCCAT) was injected on both blastomeres of two cell-stage embryos to knock down endogenous FAK expression [71]. An approximately 50% drop of FAK protein level was observed at gastrula stages but more severe reduction was seen at tadpole stages via western blotting. In morphant tadpoles staining of the intersomitic boundaries with the anti P-Y576 antibody (Figure S1E) is significantly reduced suggesting that the antibody is specific. Finally, embryo lysates were phosphatase treated and we confirmed that no protein was detected under these conditions (not shown).

### Western Blot Analysis

Protein lysates were prepared by homogenizing explants or embryos in ice cold RIPA lysis buffer (50 mM TrisHCl pH7.4, 150 mM NaCl, 2 mM EDTA, 1% NP-40, 0.1% SDS, 1% deoxycholate 24 mM) supplemented with phosphatase inhibitors (5 mM Sodium Orthovanatate, Na_3_VO_4_) and protease inhibitors (1 mM PMSF, Protease cocktail, Sigma). Homogenates were cleared by centrifugation at 15000 g for 30 min at 4°C [60]. Protein levels were determined by bicinchoninic acid assay (BCA) using the Magellan™ Data Analysis software (Tecan). The lysates were loaded on 7.5% SDS-polyacrylamide gels with the WesternC ladder (161-0376 Bio-Rad, USA). The proteins were transferred onto nitrocellulose membrane, blocked in 5% BSA (in TBST: 1X TBS & 0.1% Tween). The blotting was performed by incubation of the primary antibodies in 3% BSA, overnight at 4°C. Blots were incubated with anti-FAK mouse monoclonal (1∶200, 2A7-Upstate Biotechnology, USA), anti-FAK (1∶500, 05–537, monoclonal from Millipore, USA) or phospho-FAK antibodies, P-Y397 FAK mouse monoclonal (1∶200, MAB1144-Chemicon Millipore, USA), P-Y397 FAK rabbit polyclonal (1∶3000, ab4803-Abcam, USA), P-Y576 FAK rabbit polyclonal (1∶200, 44652G-eBiosource Invitrogen, UK), P-Y576 FAK rabbit recombinant monoclonal (1∶500, 700013-Invitrogen), P-Y861 FAK rabbit polyclonal (1∶200, 44-626G-eBiosource Invitrogen, USA), HA rabbit polyclonal (1∶500, NB600-363-Novus, UK). The incubation was performed overnight at 4°C. Visualization was performed using HRP-conjugated antibodies (1 hour incubation RT) (Santa Cruz Biotechnology anti-rabbit and anti-mouse, USA) and detected with LumiSensor (GeneScript) on UVP iBox. For loading control an actin rabbit polyclonal antibody (1∶1000, sc-1616-Santa cruz, USA) was used in every blot. Densitometry analysis was carried out using the Vision Works LS Software. The analysis of the results at Figures 1 and 5 included normalization of the intensity values of phospho-FAK signal against total FAK and averaging from three independent experiments.

### Plasmids and Cloning

All plasmids were constructed using standard molecular biology techniques and they were sequenced to verify correct coding.

#### pCS108 HA-FERM

A PCR fragment amplified with F/HA-FERM (5′-ATGCGGCCGCATGTACCCATACGATGTTCCAGATTACGCT-3′) and R/FERM (5′-TTTCTCGAGTTAATCTATTATCTCTGCATAGTCATCTGT-3′) encoding HA FERM (up to amino acids 402 including tyrosine 397), using pKH3 HA-FAK plasmid (kindly provided by Dr. Guan laboratory) as template, was inserted into the multiple cloning site of the pCS108 vector by restriction enzyme digest with NotI/XhoI.

#### pCS108 HA-FERM Y397F

A PCR fragment amplified with F/HA-FERM primer and R/FERM Y397F primer (5′-TTTCTCGAGTTAATCTATTATCTCTGCAAAGTCATCTGT-3′) encoding HA-FERM Y397F, using pKH3 HA-FAK Y397F plasmid as template was inserted into the multiple cloning site of the pCS108 vector by restriction enzyme digest with NotI/XhoI.

#### pCS2++ GFP-FRNK

GFP-FRNK construct in the adenoviral shuttle vector pShuttle was transferred to the CS2++ vector using the restriction enzymes BglII/NotI.

HA-FAKΔ375 construct (N-term 375 amino acids truncated) in pKH3 vector (kindly provided by Dr. Guan laboratory) was amplified by PCR techniques using the primers F/HA tag: 5′-ATGCGGCCGCATGTACCCATACGATGTTCCAGATTACGCT-3′and R/FRNK: 5′-TTTCTCGAGTTAGTGGGGCCTGGACTGGCTGATCATTTT-3′ and cloned into the pCS108 vector.

All mutants were generated from FAK chicken variant.

The DNA amplification reactions were performed using AccuPrime™ Pfx SuperMix (1234-040, Invitrogen, UK) which contains 22 U/ml Thermococcus species KOD thermostable polymerase complexed with anti-KOD antibodies, 66 mM Tris-SO_4_ (pH 8.4), 30.8 mM (NH_4_)_2_SO_4_, 11 mM KCl, 1.1 mM MgSO_4_, 330 µM dNTPs, AccuPrime proteins and stabilizers.

All plasmids were transcribed into RNA using mMessage mMachine Sp6 kit (Ambion, UK) and the mRNAs were purified using the Mega Clear kit (Ambion, UK).

### Imaging Analysis

Embryos were observed either under a Zeiss Axio Imager Z1 microscope, using a Zeiss Axiocam MR3 and the Axiovision software 4.8.2 or under a confocal LSM710 microscope (Zeiss, Germany). For the generation of the intensity profiles and the color coded pixel intensity profiles of the localization of the mutants the ZEN 2009 software was used.

## Supporting Information

Figure S1 **Characterization of the phosphospecific FAK antibodies.** (A) Confocal images of mid-gastrula control and Src inhibitor treated embryos stained with the phosphospecific antibodies P-Y576 and P-Y861 showing a reduction in staining intensity in the presence of the inhibitors. (B) High magnification confocal images of the focal adhesions of NIH 3T3 and FAK −/− cells stained with the P-Y576 phosphospecific antibody showing lack of FAK staining in FAK knockout cells (C) Western blot of control and FERM expressing embryo lysates blotted with the P-Y576 and P-Y861 antibodies. Membranes were stripped and reprobed with a P-Y397 antibody to visualize the phophorylated FERM. Exogenous phosphorylated FERM is not recognized by the two phosphospecific antibodies. The blot with the P-Y576 antibody has two background bands slightly above and below the size of the FERM domain (black arrowhead) but these are also present at the control lane. (D) High magnification confocal images of immunostained FERM injected embryos either with HA and P-Y397 FAK (1^st^ row) or HA and P-Y576 FAK (2^nd^ row). Colocalization analysis of these images using the Zen 2010 Software shows strong colocalization between FERM and P-Y397 indicating recognition of the Tyr397 site of the exogenously expressed FERM domain by the P-Y397 antibody but very little between FERM and P-Y576 suggesting lack of bleedthrough artifacts and crossreactivity of the antibodies. (E) Maximum Intensity Projections of confocal Z-stacks from an immunostained control and a 30 ng of FAK morpholino injected tadpole using the P-Y576 antibody. Injected tadpoles show much lower P-Y576 levels suggesting that the antibody is specific when used in whole mount immunofluorecence experiments in *Xenopus*. Western blot analysis of lysates from FAK morpholino injected and control embryos with the C-903 FAK antibody showing an aproximately 50% reduction of endogenous FAK at the gastrula stage.
(TIF)

Figure S2 Localization of the HA-FERM (A) and HA-FRNK (B) constructs in DMZ injected cells. (C) Localization pattern of P-Y397 FAK in DMZ cells.
(TIF)
