# Supplementary figures and images for: Activation of Endogenous FAK via Expression of Its Amino Terminal Domain in Xenopus Embryos
Source: PLoS One. 2012 Aug 6;7(8):e42577. doi: 10.1371/journal.pone.0042577 (PMC3412797; doi:10.1371/journal.pone.0042577)

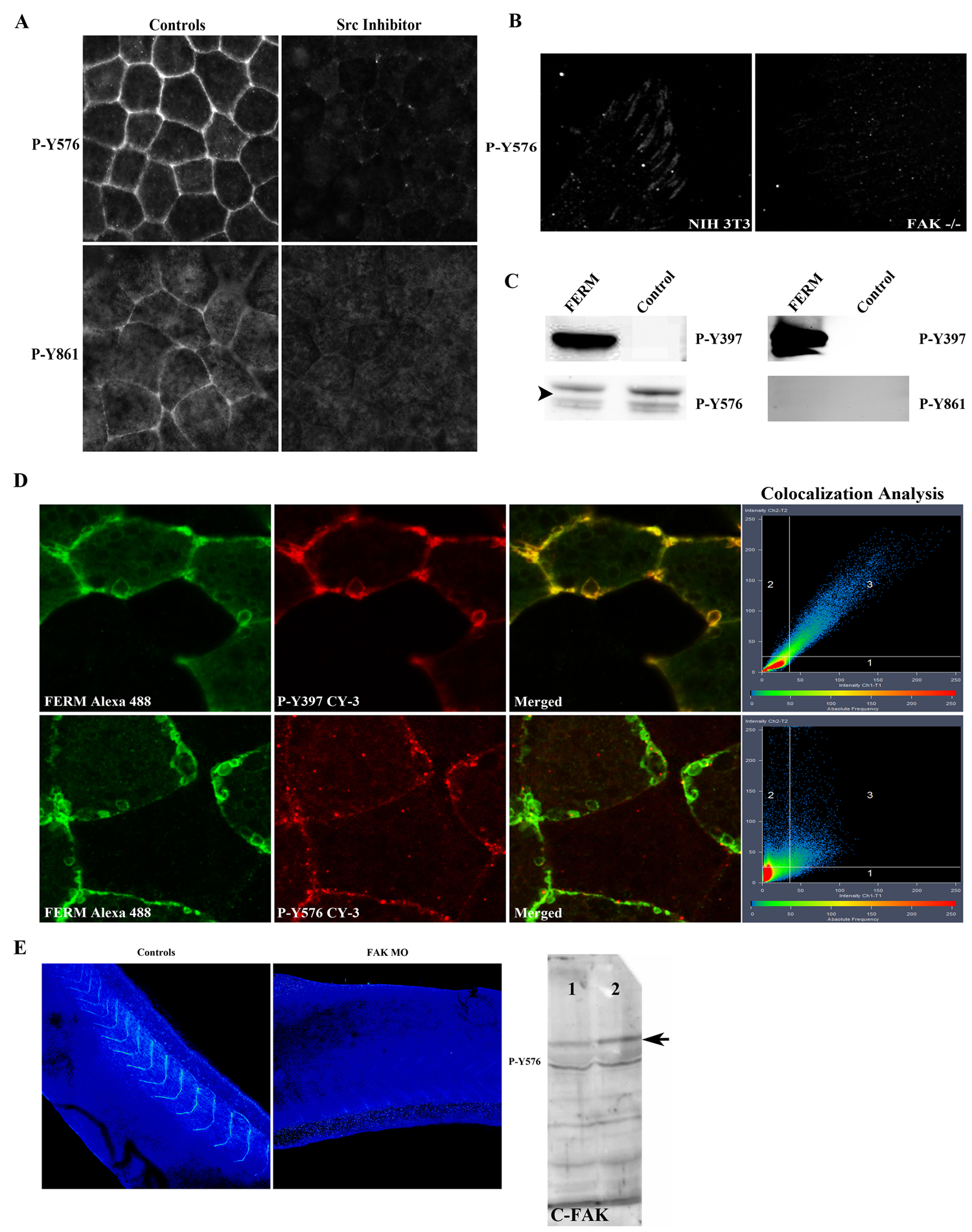

Supplement: Figure S1 — Characterization of the phosphospecific FAK antibodies. (A) Confocal images of mid-gastrula control and Src inhibitor treated embryos stained with the phosphospecific antibodies P-Y576 and P-Y861 showing a reduction in staining intensity in the presence of the inhibitors. (B) High magnification confocal images of the focal adhesions of NIH 3T3 and FAK −/− cells stained with the P-Y576 phosphospecific antibody showing lack of FAK staining in FAK knockout cells (C) Western blot of control and FERM expressing embryo lysates blotted with the P-Y576 and P-Y861 antibodies. Membranes were stripped and reprobed with a P-Y397 antibody to visualize the phophorylated FERM. Exogenous phosphorylated FERM is not recognized by the two phosphospecific antibodies. The blot with the P-Y576 antibody has two background bands slightly above and below the size of the FERM domain (black arrowhead) but these are also present at the control lane. (D) High magnification confocal images of immunostained FERM injected embryos either with HA and P-Y397 FAK (1st row) or HA and P-Y576 FAK (2nd row). Colocalization analysis of these images using the Zen 2010 Software shows strong colocalization between FERM and P-Y397 indicating recognition of the Tyr397 site of the exogenously expressed FERM domain by the P-Y397 antibody but very little between FERM and P-Y576 suggesting lack of bleedthrough artifacts and crossreactivity of the antibodies. (E) Maximum Intensity Projections of confocal Z-stacks from an immunostained control and a 30 ng of FAK morpholino injected tadpole using the P-Y576 antibody. Injected tadpoles show much lower P-Y576 levels suggesting that the antibody is specific when used in whole mount immunofluorecence experiments in Xenopus. Western blot analysis of lysates from FAK morpholino injected and control embryos with the C-903 FAK antibody showing an aproximately 50% reduction of endogenous FAK at the gastrula stage. (TIF) [file pone.0042577.s001.tif]

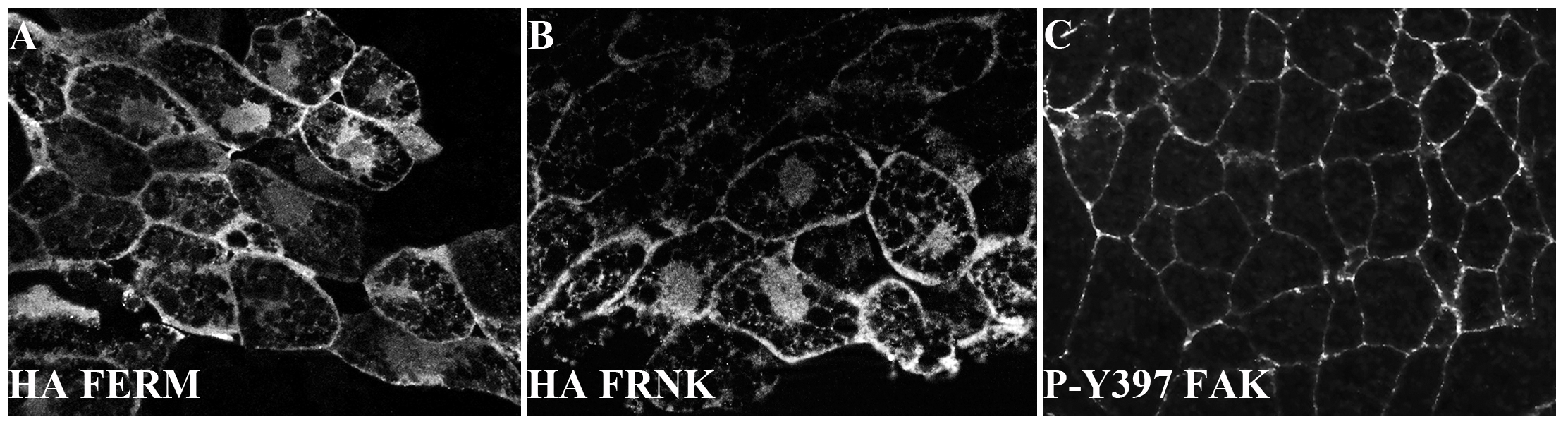

Supplement: Figure S2 — Localization of the HA-FERM (A) and HA-FRNK (B) constructs in DMZ injected cells. (C) Localization pattern of P-Y397 FAK in DMZ cells. (TIF) [file pone.0042577.s002.tif]
